# Nicotine Dosimetry in Evaluating Electronic Cigarettes Compared to Cigarette Smoking: Implications for Tobacco Regulatory Science

**DOI:** 10.1021/acs.chemrestox.4c00462

**Published:** 2025-03-17

**Authors:** Neal L. Benowitz, Hao-Yuan Yang, Peyton Jacob, Gideon St Helen

**Affiliations:** Research Program in Clinical Pharmacology, Division of Cardiology, Department of Medicine, University of California, San Francisco, Box 1237, San Francisco, California 94143, United States

## Abstract

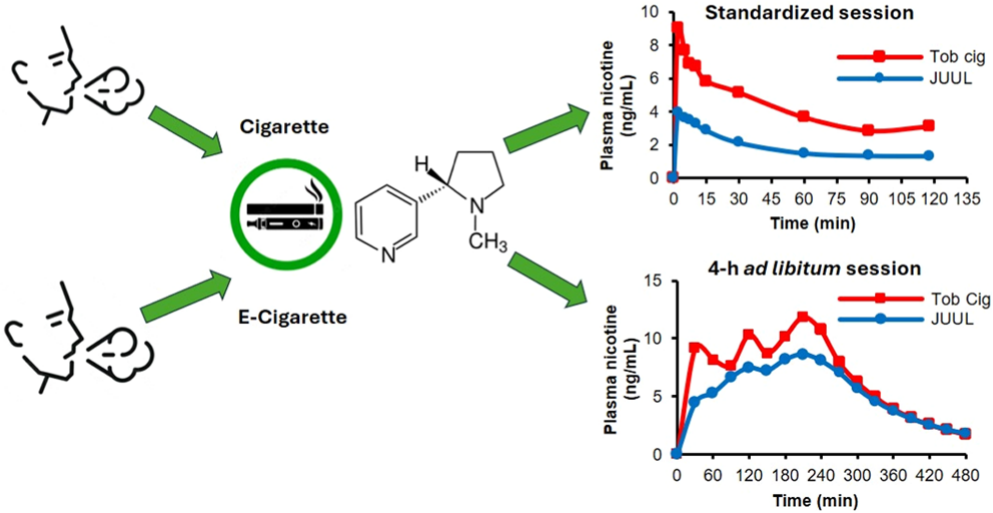

The delivery and systemic absorption of nicotine are important for assessing the potential safety and efficacy of novel inhaled nicotine delivery devices. We describe an experimental approach for examining systemic nicotine intake, looking at individual variability, comparing JUUL electronic cigarettes and cigarette smoking, and comparing standardized puffing and ad libitum use. Fourteen cigarette smokers who were infrequent e-cigarette users vaped JUUL or smoked cigarettes, both in a standardized session (ten 3.5 s puffs over 5 min) and in a 4 h ad libitum use session. Plasma nicotine concentrations were measured, and using sex and body weight-based population nicotine clearance predictions, systemic nicotine dose was estimated in each session. The pharmacokinetically (PK)-estimated nicotine dose in the standardized session averaged 0.55 mg (range 0.16–0.82) for JUUL and 1.15 mg (range 0.35–4.56) for cigarette smoking. The PK-estimated dose with ad libitum use averaged 4.1 mg (range 0.4–9.5) for JUUL and 5.0 mg (range 1.5–15) for smoking (average 3.4 cigarettes). Within individual correlations, comparing PK-estimated dose for JUUL use with standardized vs ad libitum session was weak (*r* = 0.45, NS) but was much stronger for cigarette smoking (*r* = 0.82, *p* < 0.001). Data from ad libitum use predicted that consumption of the liquid contained in a JUUL pod would correspond to smoking 15 cigarettes, which is similar to that observed in real world studies. We conclude that standardized vaping sessions do not predict usual nicotine self-administration behavior with ad libitum use. With ad libitum use, nicotine intake is much more similar to vaping and smoking and provides a much better predictor of product delivery in the real world. This approach is recommended for screening of novel inhaled nicotine devices and to aid FDA regulatory decision making.

## Introduction

Nicotine delivery, absorption, and pharmacokinetics are important features in assessing the safety and efficacy of novel nicotine delivery devices. The extent and speed of nicotine delivery and systemic absorption have implications both for benefit to aid smoking cessation and for risk, particularly abuse liability. Nicotine delivery from an electronic cigarette (e-cigarette) can be influenced by the concentration and extent of protonation of nicotine in the e-liquid and by device characteristics, such as power and the nature of the coil, which, in turn, influence aerosol particle size and distribution. Nicotine delivery is also affected by the duration, intensity, frequency, and manner of puffing and pattern of deposition of aerosol in the airways.^1,2^

A recent review of nicotine delivery and cigarette equivalents from vaping a JUUL e-cigarette reported that on average in regular users, one JUUL pod labeled as 59 mg nicotine/mL (5% by weight) delivers a nicotine dose equivalent to smoking around 18 cigarettes.^3^ This estimate was based on the measurement of urine total nicotine equivalents comparing daily smoking vs ad libitum JUUL use. However, the most common experimental approaches to dose estimation from e-cigarettes consist of standardized smoking and vaping sessions in human volunteers in whom plasma nicotine concentrations are measured in a research laboratory or machine vaping studies in which nicotine dose per puff is measured and extrapolated to the number of puffs provided by the device. In the case of JUUL, 200 puffs are reportedly produced from a JUUL pod.^3^ All of the methods yielded wide variability in predicted nicotine delivery.

We report here results of a laboratory study comparing nicotine delivery from JUUL to cigarette smoking in daily smokers who had a history of infrequent vaping to assess the potential acceptability of providing JUUL use for cigarette harm reduction and quitting in relatively novice vapers. We present an analysis of data from this study to describe a pharmacokinetic approach to nicotine dosimetry, an examination of individual variation in nicotine exposure, and an assessment of the relationships between nicotine exposure from vaping vs smoking. We discuss experimental designs that might serve as screening tools for novel nicotine delivery devices in general. In particular, we compared nicotine exposure from standardized use sessions to ad libitum use sessions.

In addition, we examined the concept of nicotine flux (nicotine dose/s), which has been proposed as a measure of nicotine delivery by a particular device or product under specified inhalational parameters.^4^ The amount of nicotine delivered per second is thought to be an important determinant of potential toxicity and abuse liability. We assessed the nicotine flux in two ways. We computed the systemic nicotine flux, as determined by pharmacokinetically estimated nicotine systemic exposure during a standardized puffing regimen for both JUUL use and cigarette smoking. For JUUL, we also computed the delivered nicotine flux, as determined by the dose of nicotine emitted per second by the device during a standardized puffing session.

## Experimental Procedures

Fourteen daily smokers of five or more cigarettes per day who had infrequently used e-cigarettes in the past (fewer than 5 days per month) were recruited by newspaper ads and the Internet. Participants had to be at least 21 years old and not intending to quit smoking in the next 3 months. Exclusions included pregnancy, using nicotine metabolism-altering medications, chronic medical disease, active substance use disorder, or recent use of illicit drugs other than marijuana. To ensure adequate regular nicotine use, a saliva cotinine concentration of 50 ng/mL or greater was required for study participation. Twenty-seven subjects were screened with exclusions for positive urine drug screen for illicit drugs, plan to quit smoking in next 30 days, high blood pressure, poor venous access, and low saliva cotinine. The study was approved by the Institutional Review Board of the University of California San Francisco. Written informed consent was obtained from each participant, and participants were financially compensated.

### Study Procedures

A two-arm counterbalanced crossover study was conducted in which participants were confined to a clinical research ward on 2 separate days. Participants were shown a training video and provided with a JUUL e-cigarette to allow experience with use of the device for one day prior to the vaping session. Each study day included a standardized vaping or smoking session in the morning, followed by a 4 h ad libitum product use session in the afternoon. On day 1, participants used a JUUL e-cigarette and on the other their usual brand of tobacco cigarette.

The order of product use was balanced. The JUUL e-cigarettes were obtained in local vape shops in tobacco or menthol flavors. None of the participants smoked menthol cigarettes as their usual brand (local menthol cigarette ban in place), but 6 participants selected menthol-flavored JUUL.

Participants were asked to abstain from tobacco product use starting at 10 pm the night before hospital admission. At approximately 8:00 am, an intravenous catheter was inserted in a forearm vein, and a light breakfast was provided. At 9:00 AM, participants used the assigned e-cigarette or their usual brand tobacco cigarette in a standardized protocol, taking one puff every 30 s for 10 puffs. This standardized use protocol has been widely used in studying nicotine delivery and acute effects of e-cigarettes.^5^ With e-cigarette use and cigarette smoking, puff duration was controlled at 3.5 s using a recorded audible signal. The 3.5 s puff duration was based on observations of people puffing JUUL e-cigarettes.^6^ Electronic cigarettes and cigarettes were weighed prior to and after the standardized use to determine the amount of liquid or tobacco consumed. Blood samples were collected before and 2, 5, 7, 10, 15, 30, 45, 60, 90, and 118 min after the last puff of each product during the standardized session to measure plasma nicotine concentrations. Heart rate, blood pressure, and skin blood flow were measured at various times after product use, and the results of which will be reported elsewhere.

After 3 h of abstinence, starting at 12:00 pm, participants vaped or smoked as desired for 4 h. During this time, participants were permitted to watch television, use computers or smartphones, and/or read books or magazines. Blood samples were obtained every 30 min prior to and until the end of the session. Electronic cigarettes and cigarettes were weighed before and after ad libitum use. Subjective questionnaires were administered before, during, and after both standardized and ad libitum sessions, and the results of which will be presented in another publication.

### Analytical Chemistry

Plasma nicotine concentrations were determined by GC-MS/MS using a modification of a published GC-MS method.^4^ Use of tandem mass spectrometry (MS/MS) improved sensitivity, providing a lower limit of quantitation of 0.2 ng/mL, and concentrating the final extract was unnecessary. The nicotine concentration of the tobacco cigarette brand smoked by each participant was extracted using a published method.^7^ Concentrations of nicotine in tobacco extracts were determined by gas chromatography with nitrogen–phosphorus detection, using 5-methyl nicotine as a standard.^8^ This method has been modified for determination using capillary GC, and the concentration of the final extract is not necessary.^9^

### Pharmacokinetic Analysis

Pharmacokinetic parameters were estimated from plasma nicotine concentrations using a Phoenix WinNonlin 6.3 (Pharsight Corporation, Mountain View, CA). Maximal plasma nicotine concentration (*C*_max_) and time to maximal concentration (*T*_max_) were measured, and the area under the plasma nicotine concentration–time curve (AUC) was computed using a noncompartmental model and trapezoidal rule for both standardized and ad libitum sessions. AUC from 0 to infinity (AUC_0–∞_) for standardized and ad libitum sessions was estimated by extrapolation from the last measured nicotine concentration using the individual participant terminal half-life that was determined in the standardized session. Since all participants had quantifiable plasma nicotine levels at baseline prior to standardized and ad libitum sessions (prestandardized, 1.3 ± 2.0 ng/mL), we corrected all measured baseline values as described previously.^10^

The PK-estimated systemic nicotine dose was computed using the AUC_(0–∞)_ and the average population clearance of nicotine (Cl) based on sex and body weight (women: 17.7 mL/min/kg; men: 16.7 mL/min/kg), using the equation: dose = Cl × AUC_(0–∞)_.^11^ We computed the systemic nicotine flux (nicotine dose/s) for each individual during the standardized session as PK-estimated systemic nicotine dose/number of puffs × 3.5 s per puff, as described by Shihadeh.^12^

### Other Data Analyses

The amount of nicotine potentially delivered by the vaping device (liquid consumption) was computed by multiplying the change in weight pre–post vaping (mg) by 50 mg nicotine/g liquid, the latter being the concentration of nicotine in the JUUL e-liquid.^3^ The amount of nicotine released from the cigarette was estimated by multiplying the weight of tobacco burned by the nicotine concentration in the filler of the particular brand smoked by the individual participant. We computed the delivered nicotine flux for JUUL using the amount of nicotine in the liquid consumed/number of puffs × 3.5 s per puff. The ratio of PK-estimated nicotine dose/delivered nicotine from JUUL as a measure of uptake fraction was also computed.

To examine relationships between the amount of product consumed and dose of nicotine absorbed, we examined within-subject Pearson correlations between the amount of nicotine in the liquid consumed from the JUUL device and between the weight of tobacco cigarette burned and nicotine content of that tobacco, with plasma nicotine AUC and PK-estimated nicotine dose. To determine how well nicotine intake during the standardized session predicts nicotine intake during ad libitum product use, we examined the within-subject correlations between nicotine AUC and PK-estimated nicotine dose, comparing the standardized and ad libitum sessions. The extent of nicotine titration comparing JUUL to cigarette smoking with ad libitum use was computed as the ratio of AUC_(0–∞)_ for [JUUL]/[cigarette], where a ratio of 1 would indicate complete titration.

## Results

The 14 participants included nine males and five females, with an average age of 32 years. Eleven were non-Hispanic white, two mixed race, and one Asian (Table 1). All were daily cigarette smokers with an average of 8.6 cigarettes per day (range 4–15). The level of cigarette dependence as estimated by the Heaviness of Smoking index averaged 1.2 (mean). All had used e-cigarettes in the past, but only two had used them in the past month. Saliva cotinine at screening averaged 96 ng/mL. The cigarette brands smoked by participants with tobacco filler weight and nicotine concentration in tobacco are shown in Table 2.

**Table 1:** Demographics and Baseline Nicotine Use Behaviors

variable	*N* (%)/mean (SD)	range
age (mean, SD)	32.1 (8)	28–37
gender		
female	5 (35.7)	
race		
non-Hispanic White	11 (78.6%)	
mixed	2 (14.3%)	
Asian	1 (7.1%)	
cigarettes per day	8.6 (4.1)	4–15
have you used e-cigarettes in the past 30 days?	yes (14.3%)	
	no (85.7%)	
heaviness of smoking index^13^	1.2	0–4
baseline saliva cotinine (ng/mL)	96.6 (62)	16.6–258.2

**Table 2:** Cigarette Brands, Tobacco Weights, and Nicotine Contents Used by Participants

cigarette brand name	*N*	weight of cigarette filler (g)	total nicotine in filler (mg)	% nicotine in filler
Camel Blue Turkish Domestic Blend	1	0.6	10.4	1.72
Natural American Spirit Mellow Taste	3	0.71	13.9	1.96
Natural American Full Bodied Taste	4	0.7	13.7	1.95
Marlboro Smooth Original Flavor Filter Cigarettes	1	0.62	9.2	1.49
Marlboro Gold Seventy Twos	1	0.58	8.2	1.41
Marlboro Sliver Seventy Twos	1	0.57	8.7	1.53
Marlboro Red Seventy Twos Class A Cigarettes	3	0.69	11	1.6

### Standardized Session

**Figure 1:**
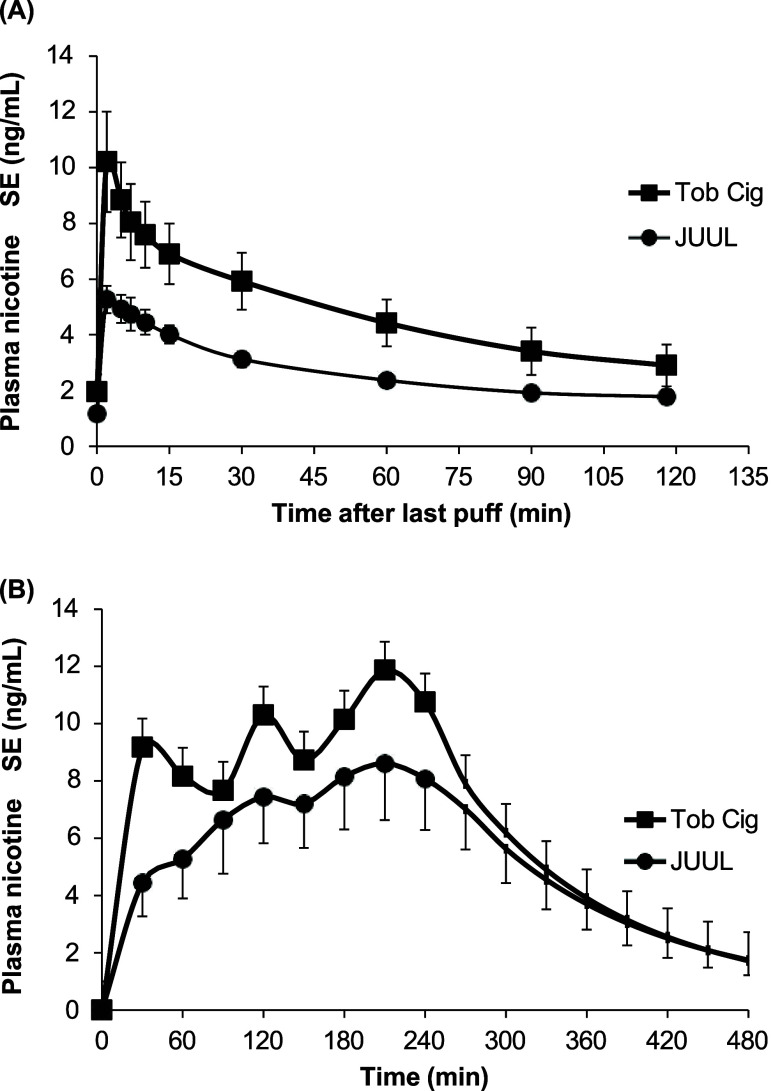
Average plasma nicotine concentrations (mean and SEM) corrected for baseline levels during the standardized session (A) and ad libitum session (B) with vaping of JUUL e-cigarette and cigarette smoking.

**Figure 2:**
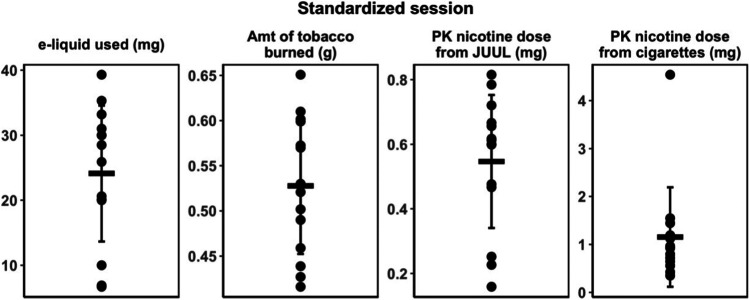
Individual variability in amount of e-liquid consumed, amount of tobacco burned, and PK-estimated nicotine dose from JUUL and cigarette smoking during standardized session. Dark bars indicate means; lighter bars indicate SEM.

Average plasma nicotine concentrations while using JUUL and usual brand cigarettes are shown in Figure 1. One participant took only 6 puffs on the JUUL and another only nine puffs from the cigarette due to a communication problem related to COVID-related safety procedures. Nicotine consumption and pharmacokinetic data during the standardized use sessions are shown in Table 3. The average weight of e-liquid consumed with the use of JUUL averaged 24 mg, with a wide range of 6.7–39.3 (Figure 2). Based on e-liquid consumption, the delivered nicotine dose averaged 1.21 mg with a range of 0.34–1.97 mg. The average tobacco burned during smoking averaged 0.53 g, with a range from 0.42 to 0.65 g (Figure 2). The estimated nicotine loss from the cigarette based on the amount of tobacco burned and the nicotine concentration in the filler averaged 9.1 mg (range 7.1–11.4).

**Table 3:** Product Consumption and Nicotine Pharmacokinetics during Standardized Session

			e-cigarette vs tobacco	
variable	e-cigarette mean ± SD (range)	tobacco mean ± SD (range)	mean ratio or difference (95% CI)	*p* value (paired *t*-test)
amount of e-liquid consumed (mg)	24 ± 10 (6.7, 39.3)			
amount nicotine delivered from JUUL(mg)	1.21 ± 0.52 (0.34, 1.97)			
amount of tobacco burned (g)		0.53 ± 0.08 (0.42, 0.65)		
amount of nicotine in burned tobacco (mg)		9.14 ± 1.29 (7.08, 11.43)		
*C*_max_ (ng/mL)	4.7 ± 2.11 (2.1, 9.7)	8.8 ± 6.31 (3.1, 23.7)	0.59 (0.27, 1.29)	0.05
*T*_max_ (min)	4.5 ± 3.9 (2, 15)	5.2 ± 4.81 (2, 15)	0.92 (0.43, 2.01)	0.63
half-life (min)	102.6 ± 67.5 (51.9, 298)	113 ± 88.9 (38.2, 396.5)	0.95 (0.44, 2.1)	0.41
baseline corrected AUC (0–240) (ng/mL min)	236.9 ± 103.27 (76.7, 392.8)	457.7 ± 344.89 (153.7, 1502.8)	0.56 (0.26, 1.22)	0.04
baseline corrected AUC (0–∞) (ng/mL min)	400.1 ± 162.6 (129.7, 689.1)	841.2 ± 766.7 (289, 3372.7)	0.54 (0.25, 1.16)	0.04
PK-estimated dose (mg)	0.55 ± 0.21 (0.16, 0.82)	1.15 ± 1.04 (0.35, 4.56)	0.54 (0.25, 1.16)	0.04
delivered nicotine flux for JUUL: delivered nicotine flux (μg/s)	35.1 ± 14.1 (9.6, 56.1)			
systemic nicotine flux (μg/s)	16.25 ± 6.1 (4.54, 23.29)	33.43 ± 29.7 (10, 129.7)	0.55 (0.25, 1.19)	0.04
ratio systemic/delivered nicotine flux (all)	0.56 ± 0.42 (0.18, 1.84)			
ratio systemic/delivered nicotine flux (excluding outlier)	0.47 ± 0.21 (0.18, 0.93)			

The average plasma nicotine Cmax after vaping JUUL (4.7 ng/mL) was lower than that seen after cigarette smoking (8.8 ng/mL) (*p* = 0.05). Similarly, nicotine AUC_(0–∞)_ was lower for JUUL vs for cigarette smoking (400 ng/mL·min vs 841 ng/mL·min) (*p* < 0.05). The PK-estimated systemic nicotine dose averaged 0.55 mg with JUUL (range 0.16–0.82) vs 1.15 mg (range 0.35–4.56) with smoking (*p* < 0.05). This corresponds to a mean per puff systemic nicotine dose of 50 μg for JUUL vs 102 μg for cigarette smoking. The within-subject correlation between weight of e-liquid consumed and the PK-estimated nicotine dose was 0.34 (NS); between weight of e-liquid consumed and Cmax was 0.41 (NS); between weight of tobacco burned and the PK-estimated dose was 0.43 (NS), while the correlation based on amount of nicotine lost from the cigarette and the PK-estimated dose was 0.42 (NS) (Table 4).

**Table 4:** Correlations between Product Consumption and Nicotine Pharmacokinetics and Dosing

variable	correlation coefficient (*r*)	*p*-value
weight of e-liquid used and PK-estimated nicotine doses for standardized session (all)	0.34	0.234
weight of e-liquid used and PK-estimated nicotine doses for standardized session (excluding outlier)	0.44	0.129
weight of e-liquid used and plasma nicotine *C*_max_ for standardized session (all)	0.41	0.143
weight of e-liquid used and plasma nicotine *C*_max_ for standardized session (excluding outlier)	0.47	0.106
weight of tobacco burned and PK-estimated nicotine doses for standardized session	0.43	0.125
weight of e-liquid used and PK-estimated nicotine doses for ad libitum session	0.778	0.001
weight of tobacco burned and PK-estimated nicotine doses for ad libitum session	0.403	0.153
the amount of nicotine in burned tobacco and PK-estimated nicotine doses for the standardized session	0.422	0.132
the amount of nicotine in burned tobacco and PK-estimated nicotine doses for an ad libitum session	0.699	0.005
plasma nicotine *C*_max_ for standardized and ad libitum sessions with JUUL	0.026	0.929
plasma nicotine *C*_max_ for standardized and ad libitum sessions with a cigarette	0.518	0.058
PK-estimated doses per cigarette smoked and nicotine in cigarette filler	0.472	0.088
PK-estimated doses for standardized and ad libitum sessions with JUUL	0.45	0.106
PK-estimated doses for standardized and ad libitum sessions with a cigarette	0.815	<0.001
PK-estimated nicotine doses comparing JUUL and smoking for the standardized session	0.367	0.196
PK-estimated nicotine doses comparing JUUL and smoking for ad libitum session	0.689	0.006

Systemic nicotine flux based on PK-estimated nicotine dose averaged 16.2 μg/s for JUUL cigarette and 33.4 μg/s for cigarette (*p* < 0.05). Delivered nicotine flux averaged 35.1 μg/s for JUUL. The fractional systemic uptake (systemic/delivered nicotine) averaged 56% for all participants, with a wide range of 0.18–1.84. The estimation in one outlier with a ratio of 1.81 (biologically impossible) was thought to be incorrect due to a technical problem in weighing the JUUL device. Excluding this individual, the average fractional uptake was 0.47 (range 0.18–0.93).

### Ad Libitum Session

**Figure 3:**
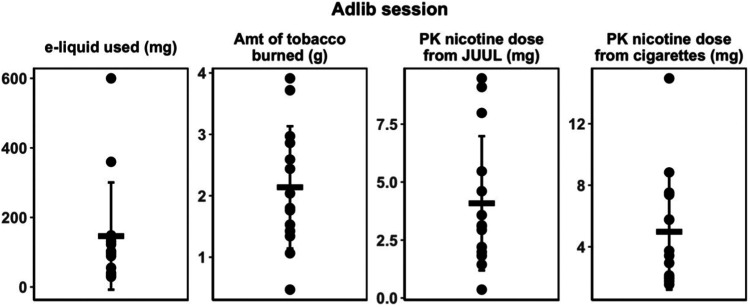
Individual variability in the amount of e-liquid consumed, amount of tobacco burned, and PK-estimated nicotine dose from JUUL and cigarette smoking during ad libitum session. Dark bars indicate means; lighter bars SEM.

Average plasma nicotine concentrations for JUUL and cigarette smoking during the 240 min ad libitum use with extrapolation over time using each participant’s half-life are shown in Figure 1. During the ad libitum vaping session, an average of 146 mg of e-liquid (range 26–900 mg) was consumed, corresponding to an average of 7.3 mg (range 1.5–30 mg) of nicotine delivered (Table 5). There was marked individual variability, as shown in Figure 3. During ad libitum smoking, participants smoked an average of 3.4 cigarettes (range 1–6) and burned an average of 2.14 g of tobacco (range 0.47–3.91 g).

**Table 5:** Product Consumption and Nicotine Pharmacokinetics during Standardized Session

			e-cigarette vs tobacco	
variable	e-cigarette mean ± SD (range)	tobacco mean ± SD (range)	mean ratio or difference (95% CI)	*p* value (paired *t*-test)
amount of e-liquid used (mg)	146 ± 154 (26, 900)			
amount of nicotine inhaled (g)	7.3 ± 7.7 (1.47, 30)			
number of cigarettes smoked		3.4 ± 1.5 (1, 6)		
amount of tobacco burned (g)		2.1 ± 0.9 (0.4, 3.9)		
amount of nicotine in burned tobacco (mg)		37.6 ± 19.3 (8.1, 76.7)		
PK-predicted nicotine dose (mg)	4.1 ± 2.9 (0.4, 9.5)	5 ± 3.8 (1.5, 15)	0.8 (0.4, 1.7)	0.3
PK-predicted nicotine dose per cigarette		1.5 ± 0.8 (0.4, 2.9)		
*T*_max_ (min)	195 ± 49 (90, 240)	154 ± 69 (30, 240)	1.4 (0.7, 3.1)	0.2
*C*_max_ (ng/mL)	11.2 ± 7.8 (1, 28.1)	15.6 ± 10 (5, 35.1)	0.7 (0.3, 1.4)	0.1
baseline corrected AUC (0–∞) (ng/mL min)	3013.7 ± 2266.2 (281.1, 8020.4)	3604.5 ± 2764.4 (1261.5, 11,113.4)	0.8 (0.4, 1.7)	0.3

**Figure 4:**
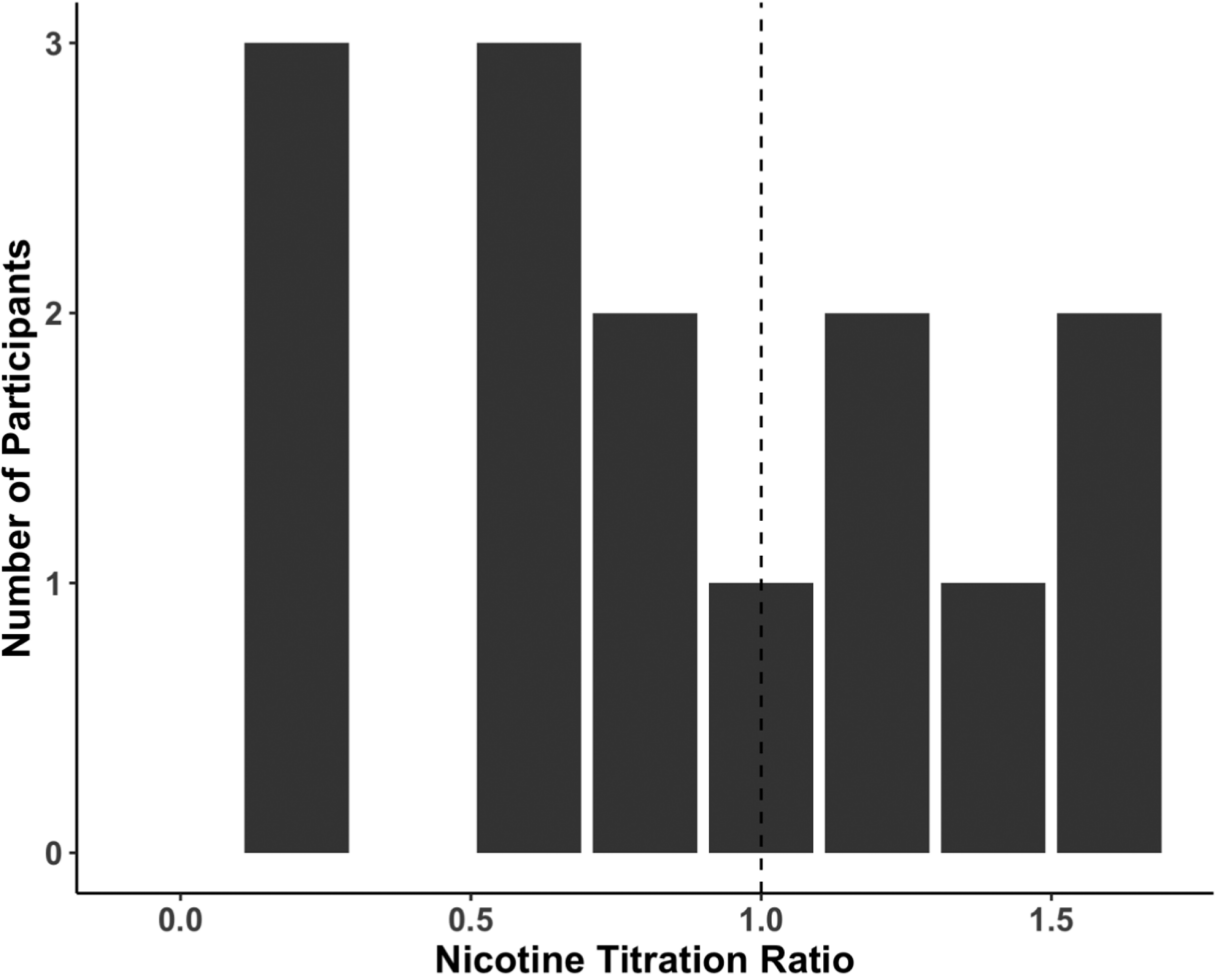
Nicotine titration ratios during ad libitum use of JUUL and cigarette smoking. Titration ratios calculated as [JUUL plasma nicotine AUC 0–∞]/[cigarette plasma nicotine AUC 0–∞] and represent the extent to which individuals titrate nicotine intake between JUUL and cigarette. The dashed line represents the point at which nicotine intake is the same for JUUL and cigarette smoking (representing complete titration).

On average, the plasma nicotine *C*_max_ (11.2 ng/mL vs 15.6 ng/mL) and AUC_(0–240)_ min were lower with JUUL use vs smoking (1641 ng/mL·min vs 2158 ng/mL·min), but these differences were not statistically significant. Nicotine AUC 0–∞ averaged 3014 ng/mL·min for JUUL and 3604 ng/mL·min for cigarette (*p* = 0.27). PK-estimated nicotine intake averaged 4.08 and 4.98 mg for JUUL and cigarette, respectively (*p* = 0.25). The within-subject correlations between weight of e-liquid used and weight of tobacco burned vs PK-estimated nicotine dose were 0.78 (*p* < 0.005) and 0.40 (NS), respectively. The correlation between nicotine in the burned tobacco filler vs PK-estimated nicotine dose was 0.70 (*p* = 0.005). The correlation between the PK-estimated nicotine dose per cigarette smoked and the amount of nicotine in the tobacco filler was 0.47 (NS). The extent of individual participant titration of nicotine intake from JUUL compared to that from cigarette smoking is shown in Figure 4. The average and median nicotine titration indices were 0.93 and 0.89, respectively.

### Relationships between Standardized and Ad Libitum Sessions

The within-subject Pearson correlations between plasma nicotine Cmax compared to standardized to ad libitum sessions were 0.026 for JUUL and 0.52 for cigarettes, respectively, both not statistically significant (Table 4). The correlations between PK-estimated nicotine dose for the standardized session and PK-estimated nicotine dose for the ad libitum session were 0.45 (NS) for JUUL and 0.82 (*p* < 001) for cigarette smoking, respectively. Thus, the strength of correlation (predictive value) for nicotine intake comparing standardized use vs ad libitum use was much stronger for cigarette smoking than for e-cigarette use.

### Estimating Equivalence of Vaping One JUUL Pod to Cigarettes per Day with Ad Libitum Use

One JUUL pod with 0.7 mL of liquid with 59 mg/mL contains 40 mg of nicotine. As noted above, on average participants consumed 7.3 mg liquid with a systemic intake of nicotine of 4.08 mg in 4 h of ad libitum use. Thus, if a person vaped 40 mg (1 pod), the systemic nicotine intake would be equivalent to 22.3 mg. Our participants smoked an average of 3.4 cigarettes, with an average systemic nicotine intake of 4.98 mg. The number of cigarettes predicted to be needed for a systemic nicotine intake equivalent to vaping one JUUL pod (22.3 mg) would be 15 cigarettes.

## Discussion

We present novel data on pharmacokinetically estimated systemic nicotine dosimetry, individual variability in nicotine dosing from e-cigarette use, cigarette smoking in relation to measures of product use, delivered, and systemic nicotine flux, and an assessment of how well measures of nicotine exposure with standardized product use predict nicotine self-administration with ad libitum e-cigarette use and smoking.

Smokers who were infrequent vapers were studied in an attempt to simulate a study of a new inhaled nicotine product that was being evaluated prior to marketing. We found that a standardized JUUL vaping session consisting of ten 3.5-s puffs, one every 30 s, resulted in wide variability in the amount of liquid consumed and in the systemic nicotine dose. The estimated systemic nicotine dose during the standardized session with JUUL averaged 0.55 mg, with a range of 0.16–0.81 mg. Plasma nicotine concentrations and PK-estimated nicotine doses were much lower with standardized JUUL use compared with standardized cigarette smoking. These

findings are consistent with other studies comparing plasma nicotine after standardized puffing of JUUL with 5% nicotine e-liquid as well as other types of e-cigarettes vs cigarette smoking.^14−17^ However, studies of experienced vapers that allowed participants to vape JUUL or smoke ad libitum for 5 min found that plasma nicotine levels were similar to JUUL 5% and cigarette smoking.^18,19^ Thus, the design of a short time-duration e-cigarette pharmacokinetic study, considering both the vaping timing and puff duration control details and the prior experience of the vaper, strongly influences results.

It has been assumed that the amount of nicotine in e-liquid consumed would approximate the systemic dose of nicotine delivered to the vaper.^20^ We found a poor correlation between the amount of nicotine contained in the liquid consumed during the standardized session and the PK-estimated nicotine intake, despite an attempt to control the puffing duration. On average we found that the fractional uptake was 47%, with wide individual variation. This discrepancy likely relates to differences in puffing intensity (air flow) and differences in the amount of nicotine exhaled and/or deposition pattern of the nicotine aerosol. Our data also suggest that some of this variability is due to differences in delivered nicotine flux across JUUL devices, as discussed in more detail below. Of note, the average PK-estimated nicotine intake per puff, 50 μg, is lower than that predicted in several vaping machine aerosol studies, but even nicotine per puff in machine vaping studies is quite variable, with a range of 72–164 μg/puff.^3^ Thus, vaping machine studies may not accurately predict the systemic nicotine intake.

With standardized puffing of a cigarette, there was considerable individual variability in the amount of tobacco burned although the extent of variation was less than that seen with the use of JUUL. The systemic nicotine intake averaged 1.15 mg, with a range of 0.35–4.0 mg, which is in the expected range for smoking a cigarette.^21^ There were weak correlations between the PK-estimated systemic nicotine dose and the weight of cigarette tobacco burned or with the amount of nicotine contained in that burned tobacco.

With ad libitum use for 4 h, the PK-estimated systemic dose from JUUL averaged 4.08 mg compared to 4.98 mg for cigarette smoking. Nicotine intake was much closer compared with the two nicotine delivery devices compared to that seen in the standardized session, with a median titration ratio of 0.89. The majority of participants titrated the nicotine dose from JUUL to 75% or more compared to that consumed from cigarette smoking. In contrast to the standardized sessions, with ad libitum use, the amount of e-liquid consumed and the amount of nicotine in the cigarette burned were significantly correlated with the systemic nicotine intake.

The use of a standardized puffing study paradigm to compare nicotine intake from vaping to cigarette smoking has been thought to be a good way to predict relative nicotine intake from the two products. The typical standardized session of 10 puffs in 5 min that we and other researchers have used is based on simulating the behavior of cigarette smokers. The difference between relative nicotine dose in standardized vs ad libitum session is likely because vapers do not puff in the same way in which they smoke cigarettes. A cigarette smoker needs to take a number of puffs in a relatively short period of time because the cigarette tobacco continues to burn. In contrast, an e-cigarette vaper spaces their puffs out according to nicotine craving over time.^22^ The hypothesis that standardized vaping sessions do not predict the usual nicotine self-administration behavior is supported by the observation that within-subject nicotine intake from vaping in the standardized session was poorly correlated with intake with ad libitum vaping. In contrast, the within-subject correlation was much stronger compared to nicotine intake from smoking in the two sessions. Despite the large difference in nicotine intake from JUUL and cigarette smoking in the standardized session, JUUL users were able to titrate their nicotine intake to a level similar to that when smoking with ad libitum use. We have made similar observations of nicotine titration with daily use of e-cigarettes compared to smoking in regular dual users.^23,24^

We were also able to examine the potential utility of the measurement of delivered nicotine flux of an e-cigarette device as a predictor of actual nicotine systemic exposure. Nicotine flux has been defined as the nicotine emitted per puff second (μg/s) from a particular device. It has been hypothesized that nicotine flux will predict nicotine delivery to the user and will predict both inadequate nicotine to satisfy smokers and high exposure to nicotine associated with high abuse liability and/or other nicotine toxicities.^12^ The determination of nicotine flux from e-cigarettes has been based on e-cigarette design, liquid composition, and puffing behavior and measured ex vivo.

We took the approach of using the in vivo measurement of nicotine released from JUUL and the PK-estimated nicotine dose to assess individual variability in delivered nicotine flux and how well-delivered nicotine flux predicts actual systemic nicotine exposure during a standardized puffing protocol. Systemic nicotine flux from both JUUL and cigarette smoking was highly variable across individuals, despite a defined 3.5 s puffing regimen. Presumably, individual differences in puffing intensity (air flow) as well as individual differences in inhalation and exhalation behavior influence systemically absorbed nicotine flux. In addition, variability in delivered flux related to the device appears to influence systemic nicotine delivery, at least for JUUL. Talih et al. determined by machine testing the nicotine delivery and flux in nine JUUL pods purchased from commercial vendors using a single JUUL device with fully charged battery.^2^ The vaping machine parameters were one 4 s puff with a flow rate of 1.5 L/min every 30 s for 10 puffs. The average nicotine emitted was 1.6 mg, and the average delivered nicotine flux was 27 μg/s mg (coefficient of variation 22%). The average nicotine delivery in the Talih study is higher than what we found (1.2 mg, coefficient of variation 42%), presumably due to the longer puff duration. The Talih study provides evidence that differences in pod performance explain some of the large individual variability in delivered nicotine flux observed in our study. Overall, our data support the idea that the nicotine flux might be useful in assessing which e-cigarette devices might not be able to deliver adequate nicotine to replace nicotine from smoking but do not inform the actual intake of nicotine related to nicotine reinforcement and abuse liability.

Limitations of our study include the relatively small participant sample size, inclusion of infrequent e-cigarette users and relatively light regular cigarette smokers, and making assumptions about individual nicotine pharmacokinetic parameters (clearance) based on population data. While our nicotine clearance estimates are based on population sex and body weight associations, nicotine clearance also varies among individuals due to other factors. While we did not explicitly determine clearance for each participant, our PK-estimates do provide a general measure of nicotine exposure and can serve as a way to compare various nicotine delivery devices. In the standardized session, a puff duration of 3.5 s was prompted by an audio signal but was not verified by direct topography measurement. The puff duration of 3.5 s was based on observations of JUUL users, but puff duration for cigarette smokers is typically shorter.^20,25^ The longer than typical puff duration with cigarette smoking could explain in part why nicotine intake was higher with smoking than with JUUL, but our estimates are consistent with nicotine intake from smoking in our studies, as described above.

The implications of our study for the methodology in assessing novel nicotine delivery devices are as follows. Using standardized use sessions to assess nicotine exposure is not a good predictor of ad libitum nicotine use for devices that are inhaled with different topographies compared to cigarette smoking. Ad libitum use sessions provide a much better indicator of expected nicotine exposure and nicotine-related effects than standardized sessions. It should be noted that during ad libitum e-cigarette use, liquid consumption does strongly predict the PK-estimated dose, which may be useful for estimating exposure when plasma nicotine concentrations cannot be measured.

While the optimal duration of use to determine nicotine equivalency of a novel device to smoking would be several days in duration, a self-administration session of even several hours can provide a reasonable estimate of device delivery in the context of its reinforcing effects.

This is illustrated by our prediction of daily equivalence of vaping one JUUL pod and cigarettes per day compared to other studies done with ad libitum product use over several days.

Our study also illustrates the limitation of trying to translate nicotine concentration in e-liquids to nicotine intake, in comparison to smoking. Because of the differences in e-cigarette device characteristics and in the composition of e-liquids, we suggest that experimental studies such as ours be used to assess nicotine dosing and abuse liability rather than basing regulation on particular e-liquid nicotine concentrations. Relatedly, there has been considerable international debate about whether concentrations of nicotine in electronic cigarettes should be capped at a particular level, in the case of the European Union at 20 mg·mL, based on typical nicotine intake from cigarette smoking.^26^ Our data suggest that assumptions used to establish nicotine limits in liquids as predictors of real-life nicotine self-administration are not valid. Nicotine intake in relation to nicotine concentration in the liquids depends on the nature of the nicotine (salt vs free base), the presence of flavors and the nature of the e-cigarette device, and inhalational behaviors and should be tested for individual e-cigarette products.^27^
